# Weight development from age 13 to 30 years and adolescent socioeconomic status: The Norwegian Longitudinal Health Behaviour study

**DOI:** 10.1007/s00038-015-0748-x

**Published:** 2015-10-07

**Authors:** Ingunn Holden Bergh, Øivind Skare, Annalena Aase, Knut-Inge Klepp, Nanna Lien

**Affiliations:** Department of Nutrition, Faculty of Medicine, University of Oslo, P.O. Box 1046, Blindern, 0316 Oslo, Norway

**Keywords:** Body mass index, Adolescents, Adults, Weight gain, Socioeconomic status

## Abstract

**Objectives:**

To describe the weight development and model change in body mass index (BMI), and to examine the association of adolescent socioeconomic status (SES) with change in BMI distribution in a cohort followed from adolescence through adulthood.

**Methods:**

Participants (*n* = 924) from western Norway were surveyed seven times from age 13 to 30 (1990–2007). BMI was based on self-reported height and weight. Quantile regression analyses were used to model change in weight development and to investigate associations between SES (measured by parental education) and change in BMI distribution. The analyses were adjusted for curvilinearity in BMI development, gender and relevant health behaviours.

**Results:**

Body mass index increased over time with the greatest increase in the 90th percentile. No significant associations between change in BMI and SES were observed at any of the percentiles (10th, 25th, 50th, 75th or 90th).

**Conclusions:**

Those in the upper BMI percentile gained more weight than those in the lower percentiles indicating that these might need targeted interventions. Further investigation of the association of change in BMI and SES with better quality data might be warranted.

## Introduction

The prevalence of overweight and obesity in children and adults in Europe is a public health concern (Berghofer et al. 2008; Brug et al. 2012). Overweight children do often become overweight or obese adults, but many develop their overweight/obesity after childhood (Power et al. 1997). In addition to gestation and early infancy and the period of adiposity rebound, adolescence is a critical periods for obesity development (Dietz 1994). While an increase in adiposity in early life may be associated with normal growth, an increase in adiposity after growth has stabilized (18–20 years) is more likely to be a threat to health (Dietz 1994; Power et al. 1997).

Norwegian studies show consistently higher levels of overweight and obesity among adults than in children and adolescents (Bjelland et al. 2010; Cuypers et al. 2012; Juliusson et al. 2010; Meyer and Tverdal 2005). The increase in overweight and obesity in recent decades seems to be a consequence of a larger weight gain over time among those with a higher BMI to begin with (Ekblom et al. 2004; Kautiainen et al. 2002). This indicates that those in the upper part of the body mass index (BMI) distribution may be more vulnerable to influences on weight gain.

An inverse relationship between socioeconomic status (SES) and body mass index (BMI) has long been recognized as a public health challenge (Ball and Crawford 2005). Among adults low SES is associated with greater weight gain than higher SES, and SES differences in weight development may differ by gender (Ball and Crawford 2005; Giskes et al. 2008). However, a differential rate of weight gain influenced by SES possibly begins early in life (Brisbois et al. 2012), and differences in adiposity and prevalence of obesity in adulthood seem to stem, at least partly, from the socioeconomic circumstances of the family (Ball and Crawford 2005; Giskes et al. 2008; Hardy et al. 2000).

Still, knowledge about the influence of adolescent SES on weight development is limited. Longitudinal studies with cohorts encompassing childhood/adolescence through adulthood with long follow-up periods have also been called for (Giskes et al. 2008; Pate et al. 2013; Tamayo et al. 2010). Furthermore, no study has investigated whether those in the upper part of the BMI distribution are more vulnerable to a possible influence of SES on weight change over time than those in the lower part of the distribution. A possible accelerated weight gain among low SES individuals in the upper part of the BMI distribution may compound existing health inequalities.

Some of the most recent longitudinal studies examining influences on weight development have provided evidence of the utility of quantile regression (Bottai et al. 2014; Mitchell et al. 2013a, b, c). In contrast to modelling the mean change, this analytic approach allows for a specific investigation of the tails of the BMI which is of particular interest in the realm of public health (Hao and Naiman 2007). Hence, the aim of this study was twofold: to describe the weight development and to model change in the BMI distribution in a cohort with young Norwegian adolescents followed into adulthood, and to examine whether adolescent SES was associated with change in the BMI distribution over time.

## Methods

### Sample

The data stem from the 17-year Norwegian Longitudinal Health Behaviour (NLHB) study among adolescents and their parents/guardians. Participants were recruited from 22 randomly selected schools in the county of Hordaland, Norway. At baseline in 1990, the sample included a representative sample of 924 students from 7th grade (78 % response rate). Questionnaires were administered in October through school at age 13–15 (1990–1992), and thereafter by mail to the participants’ home address at age 16 (1993), 18 (1995), 19 (1996), 21 (1998), 23 (2000) and age 30 (2007). A parental/guardian survey was administered in 1996. Written consent from parents/guardians and the adolescents was given prior to the study.

A detailed description of the sampling procedures and data collection in the NLHB study is presented previously (Lien et al. 2001b). The study was approved by the Norwegian Data Inspectorate. It has been conducted in accordance with ethical principles, including the provisions of the World Medical Associations Declaration of Helsinki.

### Measures

Body mass index (kg/m^2^) was calculated from the participants’ self-reported height and weight at all ages (except at age 16 when participants were not asked about this). Overweight and obesity prevalence, presented for descriptive purposes, were calculated based on International Obesity Task Force’s cut-points for the participants up until age 18 (Cole and Lobstein 2012) and by the adult World Health Organization cutoffs from age 18 (WHO 2000). The BMI data for pregnant women at age 23 (*n* = 7) and 30 (*n* = 14) (only asked for at these ages) were excluded.

The participants’ parents reported their highest level of education in 1996 (adolescents age 19). The adolescents were asked about the highest level of education for each of their parents in 1992 (adolescents age 15). The pre-coded answers were collapsed into the following categories: elementary school (no education beyond 9 years of mandatory school), upper secondary school (1–3 years of upper secondary school) and college/university (1 year or more of college/university), further labelled low, medium and high SES groups, respectively. When parental reported data for years of education were missing (42.2 %), the educational variable was supplemented with the adolescents’ response (30 %), as used previously (Lien et al. 2001a). The data from the parent with the highest reported education level or the one available were used.

Gender, soda, chocolate/sweet and breakfast consumption, physical activity and smoking habits were included as covariates. Frequency of consuming (1) sugar containing soda, (2) chocolate/sweets, and (3) breakfast was assessed by frequency questions “How often do you drink eat/drink….?” The response categories with the recoding to times per week in parentheses were for the (1) soda item: not every week (0.5); 1–2 times per week (1.5); 3–6 times per week (4.5); 1 time per day (7); greater than 1 time per day (10); for the (2) chocolate/sweets item which were assumed to be eaten more rarely: never (0); and seldom (1); 1–2 times per week (1.5); 3–6 times per week (4.5); every day (7), for the (3) breakfast item: not that often (0.5); 1–3 times per week (1.5); 4–6 times per week (5); every day (7) (Lien et al. 2001b).

Physical activity was assessed using the question: “Outside school hours (or outside work hours), how many hours per week do you do sport or exercise until you are out of breath or sweat”? The response categories with the recoding to h/week in parentheses were: none (0); about ½ h/week (0.5); about 1 h/week (1); about 2–3 h/week (2.5); about 4–6 h/week (5); 7 h or more (7) (Anderssen et al. 1995).

Smoking was assessed by the question: “How often do you smoke?” with the following response categories: every day, every week, less than once a week, and collapsed into the following ordinal levels: not smoking (1); occasional smoking (2); regular smoking (3) (Friestad and Klepp 1997).

### Statistical analyses

Descriptive statistics are presented with means and SDs for continuous variables while frequencies and percentages are used for categorical variables. Quantile regression was used for the longitudinal analyses. This approach is an extension of ordinary least square regression and models the effect of predictors across the distribution of a continuous dependent variable (Hao and Naiman 2007; Wei et al. 2006). The coefficients from the quantile regression are interpreted in the same manner as in ordinary least square regression (i.e., change in the outcome variable for each one-unit change in the predictor) (Hao and Naiman 2007). All participants having at least one BMI observation were included in the quantile regression analyses. Observations with non-complete covariate information were, however, excluded prior to analysis. In model 0, BMI was entered as the dependent variable, with study age and gender included as covariates, to describe changes in the BMI distribution over time, specified to the 10th, 25th, 50th, 75th and 90th BMI percentiles. The age variable was centred at age 13 to facilitate the interpretation of model coefficients. An interaction term between age and gender (age × gender) allowed for different linear age trends for male and females. In model 1, an age^2^ term was included to investigate if changes in BMI were linear or curvilinear, and an age^2^ × gender term was included to examine whether any curvilinearity varied by gender over time. In model 2, parental education level was added as the predictor of interest, interacting with age (age × SES), to examine if adolescent SES was associated with changes in the BMI distribution over time, keeping the covariates from model 1. Next, in model 3a, the dietary behaviours were added. In model 3b, physical activity was added. Finally, in model 3c, smoking level was added. In these three models, all added covariates were interacting with age such that the effect of each covariate could vary with age. The behavioural variables were entered step-wise to investigate whether any association between adolescent SES and changes in BMI remained when adjusting for these groups of covariates. As a last step, the moderating effect of gender in the association between SES and change in BMI (age × gender × SES) was investigated. Quantile regression assumes independent observations. As we have dependency in the date due to repeated measurements, standard deviations, and therefore also *p* values, reported from this analysis will generally be biased. Robust standard deviations were then estimated by bootstrapping using a case resampling scheme with 1000 replications (Wei et al. 2006). To retain the dependency structure in the bootstrap samples, we sampled subjects, not individual observations. This means that all observations of the sampled subjects were included in the bootstrap data.

The quantile regression analyses were conducted in Stata (version 13, College Station, StataCorp LP, Texas, USA). All other analyses were done in IBM SPSS (version 19, IBM Corp., Somers, New York, USA).

## Results

At baseline (age 13), there were slightly more boys than girls (55 vs 45 %). The proportion with low, medium and high SES was 14, 44 and 42 %, respectively. Participation rate at age 14, 15, 18, 19, 21, 23 and 30 was 96.3 % (890), 94.0 % (869), 71.0 % (656), 61.6 % (569), 60.2 % (556), 58.2 % (538), 50.3 % (465), respectively. Height and weight were reported from 80.7 % 82.8 %, 92.5, 83.2, 96.0, 91.9, 95.0, and 93.6 % at age 13, 14, 15, 18, 19, 21, 23 and 30, respectively. There were no differences between those without and with BMI at baseline and follow-up at age 14 and 15 in the gender and SES distribution, but those who were missing BMI due to either missing weight and/or height data were more likely to be boys and to belong to the low SES group at age 18, 21, 23 and 30.

Table 1 illustrates that BMI was significantly lower among females than males from age 15 and upwards. The descriptive data points to a steady increase in BMI from age 13–30 for both genders. At the last follow-up half of the men were either overweight or obese compared to about one-third of the women. Both among women and men, the descriptive data shows that less than 10 % of the sample were obese at age 30, but within the overweight/obese groups the relative proportion with obesity were higher among women (27 %) than men (16 %).

**Table 1:** Body mass index (BMI), proportion overweight/obese and obese at baseline and follow-ups in the Norwegian Longitudinal Health Behaviour study (1990–2007), all and by gender

	*n*	All	*n*	Females	*n*	Males	*p* ^a,b^
BMI, mean (SD)							
Age 13	746	18.2 (2.1)	335	18.3 (2.2)	411	18.2 (2.1)	0.487
Age 14	737	19.2 (2.2)	319	19.2 (2.2)	418	19.2 (2.2)	0.601
Age 15	804	20.1 (2.3)	359	19.9 (2.1)	445	20.3 (2.5)	0.021
Age 18	613	21.7 (2.6)	309	21.3 (2.6)	304	22.1 (2.5)	<0.001
Age 19	546	22.1 (2.6)	283	21.7 (2.8)	263	22.5 (2.4)	<0.001
Age 21	501	22.6 (2.8)	269	22.1 (2.9)	232	23.2 (2.6)	<0.001
Age 23	511	23.1 (3.3)	255	22.5 (3.5)	256	23.7 (2.9)	<0.001
Age 30	435	24.7 (3.6)	220	23.9 (3.9)	215	25.5 (3.1)	<0.001
Overweight/obese, % (*n*)							
Age 13	746	4.6 (34)	335	4.2 (14)	411	4.9 (20)	0.786
Age 14	737	5.2 (38)	319	4.1 (13)	418	6.0 (25)	0.322
Age 15	804	7.6 (61)	359	4.2 (15)	445	10.3 (46)	0.002
Age 18	613	8.5 (52)	309	6.8 (21)	304	10.2 (31)	0.172
Age 19	546	11.4 (62)	283	11.0 (31)	263	11.8 (31)	0.864
Age 21	501	16.4 (82)	269	15.2 (41)	232	17.7 (41)	0.540
Age 23	511	24.3 (124)	255	18.4 (47)	256	30.1 (77)	0.003
Age 30	435	41.6 (181)	220	32.7 (72)	215	50.7 (109)	<0.001
Obese, % (*n*)							
Age 13	746	0.3 (2)	335	NA	411	0.5 (2)	NA
Age 14	737	0.4 (3)	319	0.3 (1)	418	0.5 (2)	NA
Age 15	804	0.6 (5)	359	NA	445	1.1 (5)	NA
Age 18	613	1.0 (6)	309	0.6 (2)	304	1.3 (4)	NA
Age 19	546	1.5 (8)	283	1.8 (5)	263	1.1 (3)	NA
Age 21	501	2.0 (10)	269	1.5 (4)	232	2.6 (6)	NA
Age 23	511	3.7 (19)	255	4.3 (11)	256	3.1 (8)	NA
Age 30	435	8.3 (36)	220	8.6 (19)	215	7.9 (17)	NA

*NA* not applicable

^a^Difference between genders tested with independent *t* and Chi-square test for continuous and categorical variables, respectively

^b^Due to low numbers of obese cases, differences between gender are not statistically analysed

Table 2 shows a trend for average BMI being higher among those with low vs high SES at each age with the difference being significant at age 21 and 30. At age 14 and 18, there was a significant difference between those with medium vs high SES. There were also differences between the SES groups for the proportions of overweight/obese, with a trend for a higher prevalence among those with low vs high SES at each age (non-significant at age 19 and 23).

**Table 2:** Body mass index (BMI) and proportion overweight/obese at baseline and follow-ups by socioeconomic status (SES) in the Norwegian Longitudinal Health Behaviour study (1990–2007)

	*n* ^a^	Low SES	*n* ^a^	Medium SES	*n* ^a^	High SES	*p* ^b,c,d^
BMI, mean (SD)							
Age 13	82	18.6 (2.0)	296	18.3 (2.4)	300	18.0 (1.9)	0.059
Age 14	91	19.4 (2.1)	288	19.3 (2.5)	297	18.9 (2.0)	0.033^b^
Age 15	109	20.4 (2.3)	330	20.2 (2.4)	315	20.0 (2.1)	0.222
Age 18	65	22.1 (2.7)	237	21.9 (2.8)	277	21.4 (2.2)	0.024^b^
Age 19	57	22.5 (2.6)	212	22.2 (2.8)	251	21.8 (2.3)	0.154
Age 21	58	23.3 (3.0)	185	22.6 (3.1)	227	22.3 (2.4)	0.050^c^
Age 23	56	23.8 (3.3)	194	23.1 (3.4)	231	22.9 (2.9)	0.185
Age 30	42	25.4 (3.8)	180	25.1 (4.0)	185	23.9 (2.9)	0.003^c^
Overweight/obese, % (*n*)							
Age 13	82	4.9 (4)	296	6.8 (20)	300	2.0 (6)	0.018
Age 14	91	6.6 (6)	288	7.3 (21)	297	2.7 (8)	0.035
Age 15	109	10.1 (11)	330	9.7 (32)	315	4.1 (13)	0.014
Age 18	65	10.8 (7)	237	11.4 (27)	277	5.4 (15)	0.041
Age 19	57	17.5 (10)	212	12.3 (26)	251	8.8 (22)	0.131
Age 21	58	22.4 (13)	185	20.0 (37)	227	11.9 (27)	0.036
Age 23	56	26.8 (15)	194	25.8 (50)	231	21.2 (49)	0.461
Age 30	42	54.8 (23)	180	47.2 (85)	185	32.4 (61)	0.003

^a^ *n* for all: 678 (age 13); 676 (age 14), 754 (age 15), 579 (age 18), 520 (age 19), 470 (age 21), 481 (age 23), 407 (age 30)

^b^Difference between groups analysed by Anova for BMI; post hoc tests showed a significant lower BMI among those with high vs medium SES

^c^Difference between groups analysed by Anova for BMI; post hoc tests showed a significant lower BMI among those with high vs low SES

^d^Difference between groups analysed by Chi-square for proportion overweight/obese

Table 3 shows the results from the quantile regression analyses. In model 0, the coefficient for the age term gives the average yearly increase in BMI for males, while the coefficient for the age × gender interaction term gives the difference in yearly increase between females and males. The average increase per year in BMI over the 17-year time period in the 90th percentile was 0.54 vs 0.38 in the 10th percentiles for males, and 0.50 BMI units vs 0.23 for females. This corresponds to a total increase in BMI over the 17-year period of 9.17 in the 90th percentile vs 6.47 in the 10th percentile for males and of 8.47 vs 3.90 for females.

**Table 3:** Changes in the body mass index (BMI) distribution across the 10th, 25th, 50th, 75th and 90th percentile and the influence of socioeconomic status (SES) in the Norwegian Longitudinal Health Behaviour study (1990–2007) estimated by quantile regression

	Body mass index (BMI): all									
	10th percentile coefficient, (95 % CI)	*p*	25th percentile coefficient, (95 % CI)	*p*	50th percentile coefficient, (95 % CI)	*p*	75th percentile coefficient, (95 % CI)	*p*	90th percentile coefficient, (95 % CI)	*p*
Model 0^a^										
Intercept	16.52 (16.36, 16.68)	<0.001	17.56 (17.43, 17.70)	<0.001	18.74 (18.53, 18.94)	<0.001	20.26 (20.00, 20.52)	<0.001	21.78 (21.24, 22.32)	<0.001
Gender (female vs male)	0.03 (−0.24, 0.30)	0.84	0.00 (−0.28, 0.30)	0.96	0.09 (−0.22, 0.40)	0.57	0.04 (−0.36, 0.44)	0.85	−0.12 (−0.79, 0.55)	0.73
Age	0.38 (0.36, 0.40)	<0.001	0.41 (0,39, 0.43)	<0.001	0.46 (0.43, 0.49)	<0.001	0.48 (0.45, 0.51)	<0.001	0.54 (0.45, 0.63)	<0.001
Age × gender (female vs male)	−0.15 (−0.19, −0.11)	<0.001	−0.15 (−0.18, −0.11)	<0.001	−0.15 (−0.19, −0.10)	<0.001	−0.08 (−0.14, −0.03)	0.005	−0.04 (−0.15, 0.07)	0.47
Model 1^a^										
Intercept	16.20 (16.00, 16.40)	<0.001	17.06 (16.84, 17.29)	<0.001	18.12 (17.90, 18.34)	<0.001	19.53 (19.33, 19.74)	<0.001	21.18 (20.74, 21.62)	<0.001
Gender (female vs male)	−0.13 (−0.45, 0.20)	0.45	0.00 (−0.29, 0.28)	0.98	0.25 (−0.08, 0.57)	0.13	0.28 (−0.15, 0.71)	0.20	0.00 (−0.63, 0.64)	1.00
Age	0.65 (0.57, 074)	<0.001	0.78 (0.70, 0.85)	<0.001	0.83 (0.07, 0.90)	<0.001	0.85 (0.76, 0.93)	<0.001	0.89 (0.75, 1.03)	<0.001
Age^2^	–0.02 (–0.02, –0.01)	<0.001	–0.03 (–0.03, –0.02)	<0.001	–0.03 (–0.03, –0.02)	<0.001	–0.02 (–0.03, – 0.02)	<0.001	–0.03 (–0.04, –0.02)	<0.001
Age × gender (female vs male)	–0.10 (–0.22, 0.02)	0.098	–0.25 (–0.35, –0.15)	<0.001	–0.24 (–0.35, –0.14)	<0.001	–0.20 (–0.32, –0.07)	0.003	–0.15 (–0.37, 0.07).	0.183
Age^2^ × gender (female vs male)	–0.00 (–0.01, 0.00)	0.329	0.01 (0.00, 0.01)	0.015	0.01 (0.00, 0.01)	0.042	0.01 (–0.00, 0.01)	0.101	0.01 (–0.00, 0.03)	0.075
Model 2^b,c^										
Intercept	16.43 (16.00, 16.86)	<0.001	17.22 (16.74, 17.70)	<0.001	17.62 (16.83,18.41)	<0.001	19.95 (19.46, 20.44)	<0.001	21.12 (20.00, 22.24)	<0.001
Age × SES (medium vs low)	–0.04 (–0.13, 0.05)	0.395	–0.04 (–0.11, 0.04).	0.378	–0.03 (–0.10, 0.04)	0.421	–0.01 (–0.13, 0.11)	0.871	–0.02 (–0.20, 0.16)	0.812
Age × SES (high vs low)	–0.02 (–0.11, 0.06)	0.605	–0.04 (–0.12, 0.03)	0.276	–0.03 (–0.10, 0.05)	0.473	–0.06 (–0.19, 0.06)	0.302	–0.15 (–0.32, 0.01)	0.072
Model 3^b,d^										
Intercept	16.23 (15.62, 17.10)	<0.001	17.62 (16.83, 18.41)	<0.001	19.40 (18.57, 20.24)	<0.001	20.78 (19.87, 21.68)	<0.001	22.54 (21.34, 23.73)	<0.001
Age × SES (medium vs low)	–0.07 (–0.16, 0.02)	0.148	–0.03 (–0.10, 0.04)	0.421	–0.01 (–0.10, 0.07).	0.880	–0.04 (–0.16, 0.09)	0.555	–0.02 (–0.20, 0.15)	0.799
Age × SES (high vs low)	–0.05 (–0.14, 0.04)	0.235	–0.03 (–0.10, 0.05)	0.473	–0.05 (–0.13, 0.03)	0.242	–0.08 (–0.21, 0.05)	0.211	–0.16 (–0.32, 0.01)	0.072

^a^In Model 0 and 1: 4893 observations/908 individuals, age (values: 0, 1, 2, 3, 5, 6, 8, 10, and 17)

^b^In Model 2 and 3: 4280 observations/810 individuals. To compare model 2 and 3 for the same number of observations, only observations with complete BMI and covariate information were included

^c^Model 2 were adjusted for age, age^2^, gender, age × gender, age^2^ × gender, SES; data not shown

^d^Model 3 were adjusted for age, age^2^, gender, age × gender, age^2^ × gender, SES, dietary, physical activity and smoking behaviour; data not shown

Model 1 shows that the change in BMI was curvilinear for males (age^2^) in all the BMI percentiles and the rate of change decelerated with increasing age. The deceleration in the rate of change was smaller among females (age^2^ × gender) in the 25th, 50th and 90th percentile (borderline significant). The coefficient for the age term gives the initial increase at age 13 in BMI per year for males. In model 1, a higher rate of initial increase in BMI per year was observed in the upper vs lower tail of the distribution with increasingly larger coefficients (age) for each percentile. For example at the 90th BMI percentile the initial increase for males was 0.89 (95 % CI 0.75, 1.03) BMI-units per year (age) compared to 0.65 (95 % CI 0.57, 07.4) at the 10th percentile. The rate of initial increase in BMI per year was significantly lower in females (age × gender) than males in each percentile, except for the lowest and upper most percentile.

**Fig. 1:**
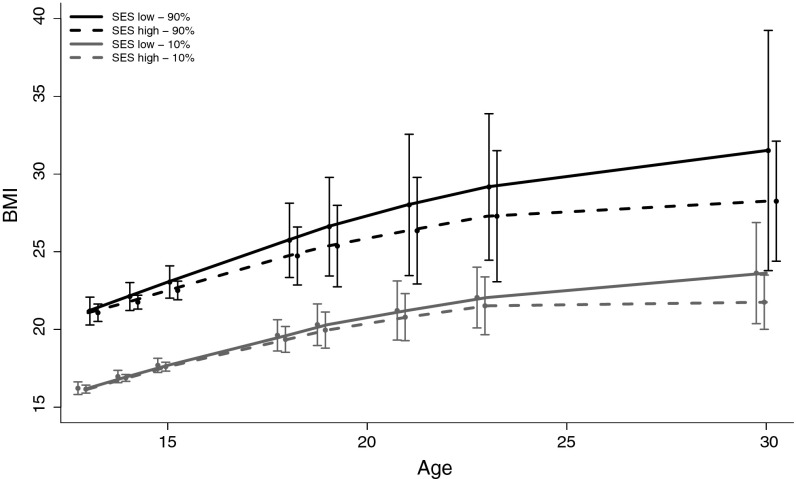
10th and 90th percentiles of body mass index (BMI) for low and high socioeconomic status (SES) in the Norwegian Longitudinal Health Behaviour study (1990–2007). The BMI percentile values are based on estimates from quantile regression (model 3)

Model 2 shows that there were no significant association between SES and change in BMI. However, the association was strongest in the upper most percentile (90th); predicted increase in BMI of [−0.15 (95 % CI −0.32, 0.01), *p* = 0.072]. There were no substantially different results between model 3a, b and c, therefore, only the fully adjusted model (numbered model 3), including the complete set of the behavioural covariates, is presented in Table 3. The strongest association between SES and change in BMI at the 90th percentile observed in model 2 remained, indicating a smaller increase in BMI per year [−0.16 (95 % CI −0.32, 0.01) *p* = 0.07] having a high vs low SES. Figure 1 illustrates the relationship between SES and change in BMI at the 10th and 90th percentile over time. Gender did not moderate any associations between SES and change in BMI in any of the percentiles.

## Discussion

The main results from this study indicate a pattern of continuous increase in average BMI over time in both genders. However, at age 30, the proportion of overweight including obese was substantially higher among males than females. Social gradients at age 30 for both average BMI and the overweight/obesity prevalence were observed. The quantile regression analyses demonstrate that both the initial and average increase in BMI over time were higher in the upper vs lower percentiles, but there were no significant association between SES and change in BMI in any parts of the BMI distribution.

The Young Hunt study from Nord Trøndelag County in mid-Norway compared two cohorts 30 years apart. They found a prevalence of overweight including obesity among adolescents (age 14–18) of 18 % in the last cohort (1995–1997) compared to 10 % 30 years earlier (Bjornelv et al. 2009). Height and weight were measured objectively and somewhat later than the adolescence data in the NLHB study, which may partly explain the lower prevalence rates seen in our study. However, in the Hunt3 study, conducted in 2006–2008 among adults (*n* ≈ 5000), 49 % of the men age 20–29 were overweight or obese (Midthjell et al. 2013). This is quite comparable to the estimate of 51 % among the 30-year-old men in NLHB study. The corresponding number for women in the Hunt3 study was 39 % (Midthjell et al. 2013), a somewhat higher estimate than the 32 % seen in our study. Parental data from 2007 from a large intervention study in the Southeastern part of Norway shows, in line with our study, an overweight/obesity prevalence based on self-report of 30 % among women, and a somewhat higher prevalence of 59 % among men (mean age 41 and 43, respectively). Taken together, our study and previous Norwegian data point to an increase in BMI taking place after the adolescence years, with evidence for a substantial increase happening throughout young adulthood (Cuypers et al. 2012).

Furthermore, when modelling change in BMI in our study, the change in BMI was greatest among those in the highest percentile, which suggests that the largest weight gain is occurring among those with the highest BMI. Even though we saw a weight gain over time in all percentiles, the result supports that the increase in overweight and obesity in recent decades may be a consequence of changes in the upper end of the BMI distribution (Ekblom et al. 2004; Kautiainen et al. 2002).

Our descriptive data indicated a development of a social gradient between the low and high adolescent SES groups over time, while the quantile regression analyses showed no significant association between adolescent SES and change in weight development across the BMI distribution. Hardy et al. (2000) examined the association between childhood SES and change in BMI during adult years. They found a relationship between father’s occupation and weight gain, which increased from age 20 to 43. Thus, there might have been evidence for the influence of adolescent SES on change in BMI in our study if the cohort had been followed up longer into adulthood. Another European study also found an association between father’s occupation and change in BMI during adulthood, but for women only (Giskes et al. 2008). In our study, however, the association between adolescent SES and change in BMI did not vary by gender in any part of the BMI distribution.

Results from other longitudinal studies among adults examining SES and change in BMI show more consistent results when using “occupation” as an indicator vs “education” (Ball and Crawford 2005). However, parental education level was used as the SES indicator in this study as it seems to be a better indicator than parental income in western developed countries (Shrewsbury and Wardle 2008). It shows the strongest association with weight status among Scandinavian adolescents (Juliusson et al. 2010; Lien et al. 2007; Matthiessen et al. 2014), and is assumed to stay relatively stable over time. A large 11-year study among adults (age 20–79) from two regions in Norway, conducted about the same time period as our study (1990–2001), showed in consistence with the findings in the NLHB study that weight gain occurred across all education and income brackets with no differential associations between SES and change in BMI (Reas et al. 2007).

There may be several explanations for our finding of no association between adolescent SES and weight development. The rapid development of an obesogenic environment over the last decades could possibly have contributed to a narrowing of the socioeconomic gap in the BMI distribution (Zhang and Wang 2004). Another hypothesis suggests that education level in general may be more strongly related to weight and obesity prevalence when other socioeconomic resources are low (Mirowsky and Ross 2003). In contradiction, Norway is a relatively high-income country with relative small average difference between those “less and better well off”.

The long follow-up period with participants from late childhood to middle adulthood is the main strength of this study. In addition, the parental education measure was based on reports taken during the adolescent years and should not be affected by recall bias to the same extent as in other studies using retrospective recall (Galobardes et al. 2006; Giskes et al. 2008). However, the predictive value of the SES variable may have been reduced because it was constructed from both parents’ and adolescents’ reports of parental educational level, and the congruence between adolescents’ and parents’ report has previously been found to only be fair (Lien et al. 2001a). In addition, some parents may have improved their education level between the start of the study in 1990 and 1996, when they reported their education level. Males and those with low adolescent SES were underrepresented due to non-response after age 18. Self-reported height tends to be over-reported and weight under-reported, especially among the heaviest and those adolescents who regard themselves as more fat (Connor Gorber et al. 2007; Flood et al. 2000; Jansen et al. 2006). Therefore, the BMI levels and overweight/obesity prevalences are probably underestimated in our study. Among adolescents underestimation is found to be higher in low SES groups as well (Jansen et al. 2006). Hence, biases related to both drop-out and self-reported BMI may have caused an attenuation of the association between SES and change in BMI. Furthermore, we have not been able to adjust for the participants’ own education level as adults because the decline in participation by age 30 caused a high number of missing for this variable. Including two probably highly collinear variables to a regression model could, however, lead to invalid conclusions (Galobardes et al. 2006). We recommend that further studies should examine both childhood and adulthood SES, and also assess whether change in SES throughout the life course influences weight development.

In conclusion, despite the limitations, this study indicates that those in the upper BMI percentile are those at most risk of gaining weight and might be more prone to the influence of SES on weight gain over time. Targeted strategies to reach those with the highest risk of developing an unhealthy weight seem needed, while inequalities in weight gain should be further explored with better measures of both BMI and SES.
